# Smoking and Risk of Prosthesis-Related Complications after Total Hip Arthroplasty: A Meta-Analysis of Cohort Studies

**DOI:** 10.1371/journal.pone.0125294

**Published:** 2015-04-24

**Authors:** Songsong Teng, Chengqing Yi, Christian Krettek, Michael Jagodzinski

**Affiliations:** 1 Department of Orthopedic Trauma, Hannover Medical School, Hanover, Germany; 2 Department of Orthopedics, Shanghai First People's Hospital, Shanghai Jiao Tong University, Shanghai, P. R. China; 3 Department of Orthopedic Trauma, Agaplesion ev. Hospital Bethel, Bückeburg, Germany; National Cancer Center, JAPAN

## Abstract

**Objective:**

Increasing evidence suggests that smoking may increase the incidence of prosthesis-related complications after total hip arthroplasty (THA). We performed a meta-analysis of cohort studies to quantitatively evaluate the association between smoking and the risk of prosthesis-related complications after THA.

**Methods:**

Relevant articles published before August 15, 2014, were identified by searching the PubMed, EMBASE and Cochrane library databases. Pooled risk ratios (RRs) or weighted mean differences (WMDs) with 95% confidence intervals (CIs) were calculated with either a fixed- or random-effects model.

**Results:**

Six cohort studies, involving a total of 8181 participants, were included in the meta-analysis. Compared with the patients who never smoked, smokers had a significantly increased risk of aseptic loosening of prosthesis (summary RR=3.05, 95% CI: 1.42-6.58), deep infection (summary RR=3.71, 95% CI: 1.86-7.41) and all-cause revisions (summary RR=2.58, 95% CI: 1.27-5.22). However, no significant difference in the risk of implant dislocation (summary RR= 1.27, 95% CI: 0.77-2.10) or length of hospital stay (WMD=0.03, 95% CI: -0.65-0.72) was found between smokers and nonsmokers.

**Conclusions:**

Smoking is associated with a significantly increased risk of aseptic loosening of prosthesis, deep infection and all-cause revisions after THA, but smoking is not correlated with a risk of implant dislocation or the length of hospital stay after surgery.

## Introduction

Total hip arthroplasty (THA) is a highly effective treatment for end-stage osteoarthritis or rheumatoid arthritis of the hip joints when conservative therapy has failed because it contributes to excellent pain relief and great improvement in the hip joint function and patient quality of life [1,2]. However, postoperative prosthesis-related complications, such as aseptic loosening, infection and dislocation, among others, usually lead to the failure of THA, which necessitates the investigation of relevant risk factors.

Recently, various investigations have demonstrated that patients' own lifestyles may impact the outcomes of elective orthopedic surgery; among them, smoking has been widely accepted as an important risk factor for the development of postoperative complications [3–5]. It was reported that patients who smoke have a longer surgical and anesthetic time, longer stay in the hospital and higher charges [6]. According to a randomized study performed by Moller et al. [7], the overall complication rate in patients who underwent total joint arthroplasty and received a smoking cessation intervention significantly decreased by 34% (p = 0.0003) compared to the patients who continued smoking. Additionally, Kapadia et al. [8] reported that smokers had much higher revision rates than nonsmokers after total knee arthroplasty.

Although several published investigations have identified the association between smoking and the incidence of prosthesis-related complications after THA, the outcomes have been inconsistent [9–14]. For example, Kapadia et al. [9] reported a lack of association between smoking and aseptic loosening of a prosthesis, whereas Meldrum et al. [14] reported a possible positive association. Moreover, the number of the cases in each study was relatively small. We performed this meta-analysis to quantitatively assess the association between smoking and the risk of prosthesis-related complications after THA from cohort studies.

## Methods

### Search strategy

This meta-analysis was conducted according to the Meta-Analysis of Observational Studies in Epidemiology (MOOSE) guidelines [15]. We systematically searched PubMed, EMBASE and the Cochrane Library databases using key terms (total hip arthroplasty OR total hip replacement) AND (smoking OR tobacco) on August 15, 2013. The search strategies are summarized in Table 1. The reference lists of all retrieved articles were also reviewed to identify additional relevant studies.

**Table 1:** Search strategy for PubMed on August 15, 2014.

Search strategy	Search terms
#1	smoking
#2	tobacco
#3 #1 OR #2	
#4	total hip arthroplasty
#5	total hip replacement
#6 #4 OR #5	
#7 #3 AND #6	

### Study selection criteria

Two independent reviewers first screened the titles and abstracts to identify the relevant investigations. Then, full articles were read to include the eligible studies that met the following criteria: (1) used a cohort study design, (2) evaluated the association between smoking and the risk of any prosthesis-related complication after THA, and (3) provided sufficient data for calculating the risk ratio (RR) or weighted mean difference (WMD) with a 95% confidential interval (CI). Reviews, editorials, letters, conference abstracts and studies that did not report the necessary information were excluded.

### Data extraction and quality assessment

For every eligible study, the following data were extracted by two independent reviewers: surname of the first author, publication date, study location, study population characteristics, total number of patients, follow-up duration, number of patients who had prosthesis-related complications and who required revisions, length of hospital stay after THA, controlled confounders and methods used for controlling the confounders.

The quality of all included studies was assessed based on the Newcastle-Ottawa Scale (NOS), a validated tool for assessing the quality of observational studies [16]. Studies with scores of 0–3, 4–6 and 7–9 were regarded as low, moderate and high quality, respectively. Only studies with adequate quality (NOS Score>2) were included.

### Statistical analysis

The WMD with a 95% CI was calculated for continuous outcomes, and RR with a 95% CI was carried out for dichotomous outcomes. As some studies did not report the mean and standard deviation for the length of hospital stay, the methods suggested by Greenland [17] were utilized to estimate these parameters from the published data. In the studies reporting the median and quartiles, the mean and standard deviation could be calculated based on the approaches described by Whitlock et al. [18].

Heterogeneity across investigations was evaluated using the Cochrane Q test and I^2^ statistic. If p<0.1 or I^2^ >50%, the heterogeneity was considered statistically significant and a DerSimonian and Laird random-effects model was utilized to combine the data; otherwise, a Mantel-Haenszel or inverse-variance fixed-effects model was used to calculate the pooled RR or WMD among studies. Sensitivity analysis was conducted by omitting one study in each turn and pooling the data of the remaining studies to explore the possible explanations for high heterogeneity and to determine the stability of the outcomes. The publication bias was not evaluated because the number of involved studies was too small (n<10) [19]. All analyses were performed using RevMan version 5.3 (Cochrane Collaboration, Oxford, UK). A p-value < 0.05 was considered statistically significant.

## Results

### Literature search

**Fig 1:**
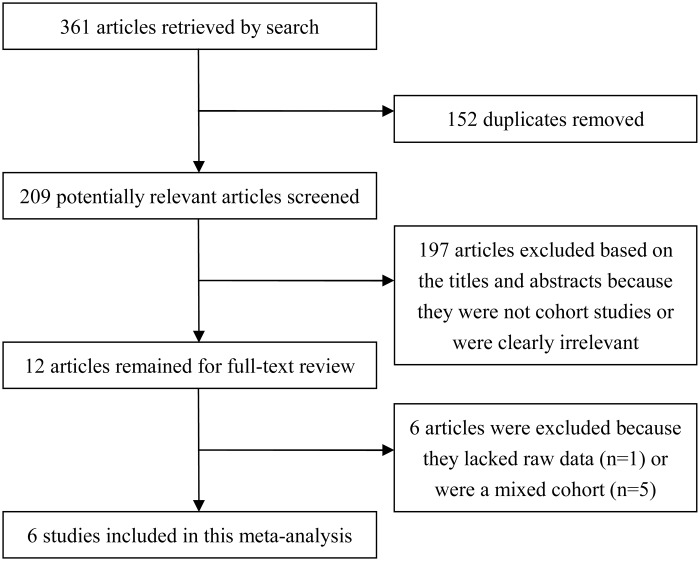
Flow chart of the assessment and selection of publications for the meta-analysis.

A flow diagram showing the study selection process is presented in Fig 1. A total of 361 articles were identified by the search strategy. After removing 152 duplicates, 209 articles remained for further screening of titles and abstracts. Afterwards, 197 articles were excluded because they were not cohort studies or were clearly irrelevant. After evaluating the full text of the remaining 12 publications, 6 articles were excluded because they had 'mixed cohort' (n = 5) or 'no raw data were reported' (n = 1). Finally, 6 studies were included in the meta-analysis.

### Study characteristics

The primary characteristics of the 6 cohort studies are summarized in Table 2. These studies were conducted in the United States (n = 3), Sweden (n = 2) and United Kingdom (n = 1), and a total of 8181 participants with 4549 smokers and 3632 nonsmokers were included in the meta-analysis. Among them, two studies only consisted of males. The duration of follow-up ranged from 2 months to 13 years, and the mean ages of the patients were between 60 and 70 years old. The reported prosthesis-related complications mainly included aseptic loosening (n = 3), deep infection (n = 4) and implant dislocation (n = 3), and 3 studies also reported the length of hospital stay after THA. The control of confounding factors differed across studies. The main controlled factors were age, gender and body mass index (BMI), and the methods used included matching, restriction and multivariate analysis (Table 2). Moreover, based on the quality assessment of NOS, 5 studies were high quality and 1 study was moderate quality (S1 Table). All 6 studies had adequate quality for meta-analysis.

**Table 2:** Characteristics of the studies included in the meta-analysis.

Authors and year of publication	Country	Study design	Number of patients	Duration of follow-up	Mean age(years old)	Male (%)	Prosthesis-related complications reported	Controlled confounders	Methods used for controlling confounders	Quality score
Kapadia et al. [9], 2014	United States	Retrospective	Smokers 110 Nonsmokers 220	Mean 4.25 years	60.4	44.9	Aseptic loosening, infection, instability and pain	Age, gender, BMI, surgery date and follow-up	Matching	7
Lombardi et al. [10], 2013	United States	Retrospective	Smokers 256 Nonsmokers 271	Mean 2.67 years	64	43	Aseptic loosening, infection, dislocation, periacetabular fracture and liner breakage	None of the potential confounding factors such as age, gender, BMI and history of infection, diabetes and cardiac disease differed between the failure and nonfailure groups	None	7
Khan et al. [11], 2009	United Kingdom	Prospective	Smokers 850 Nonsmokers 917	Up to 5 years	69	35.9	infection	Age, gender, pre-operative HHS, arteriosclerosis and transfusion requirements	Multivariate analysis for the length of hospital stay	8
Azodi et al. [12], 2008	Sweden	Prospective	Smokers 1273 Nonsmokers 833	Up to 8 years	Not reported	100	dislocation	Age, gender, BMI, history of previous THA, rheumatic disease, secondary osteoarthritis, lower extremity fractures, previous orthopedic surgical procedures from the lower back to the ankle joint, alcohol or drug abuse, fixation principle, and calendar period	Restriction, Multivariate analysis for the risk of dislocation	9
Azodi et al. [13], 2006	Sweden	Retrospective	Smokers 2029 Nonsmokers 1275	Up to 2 months	Not reported	100	Overall rate of local complications	Age, gender, BMI, history of previous THA, rheumatic disease, secondary osteoarthritis, lower extremity fractures, previous orthopedic surgical procedures from the lower back to the ankle joint, calendar period, medical region, diabetes, congenital heart failure, chronic obstructive lung disease and a history of cerebrovascular or acute myocardial events	Restriction, Multivariate analysis for the length of hospital stay	7
Meldrum et al. [14], 2005	United States	Retrospective	Smokers 31 Nonsmokers 116	Mean 13 years	60.5	46.1	Aseptic loosening, infection, dislocation and osteolysis	Age gender, BMI, diagnosis, stem fixation and alcohol use	Multivariate analysis for the risk of aseptic loosening	6

BMI: body mass index; HHS: Harris hip score; THA: total hip arthroplasty.

### Aseptic loosening of hip prosthesis

**Fig 2:**
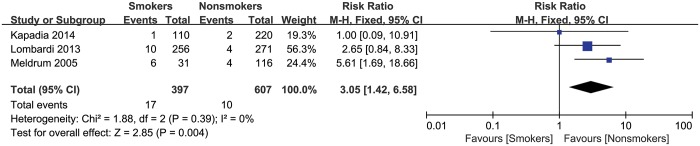
Forest plot of the association between smoking and the risk of aseptic loosening of prosthesis after total hip arthroplasty.

The presence of aseptic loosening of the hip prosthesis was reported in 3 studies with a total of 1004 patients. No heterogeneity was found among these studies (I^2^ = 0%). We found that smokers had a significantly increased risk of aseptic loosening of hip prosthesis compared to those who never smoked (summary RR = 3.05, 95% CI: 1.42–6.58) (Fig 2).

### Deep infection

**Fig 3:**
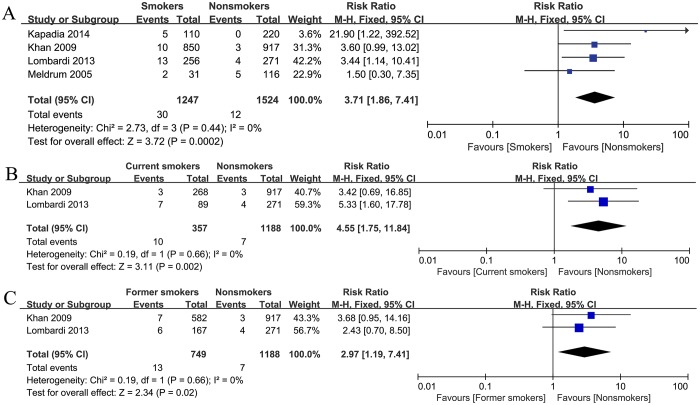
Forest plots of the association between smoking (A), current smoker (B), former smoker (C) and the risk of deep infection after total hip arthroplasty.

Deep infection was mentioned in 4 studies, including a total of 2771 patients. There was no heterogeneity among these studies (I^2^ = 0%). Meta-analysis of the included studies suggested a significantly increased risk of deep infection in the smokers after THA (summary RR = 3.71, 95% CI: 1.86–7.41). We also pooled the RR of deep infection in 2 studies that reported data on current smokers, former smokers and nonsmokers, respectively. Compared with the patients who never smoked, both current smokers (summary RR = 4.55, 95% CI: 1.75–11.84) and former smokers (summary RR = 2.97, 95% CI: 1.19–7.41) had a significantly higher risk of deep infection (Fig 3).

### Implant dislocation

**Fig 4:**
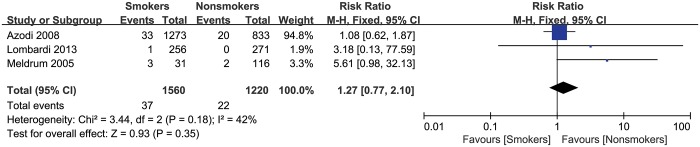
Forest plot of the association between smoking and the risk of implant dislocation after total hip arthroplasty.

Three studies, including a total of 2780 patients, reported the number of patients who experienced implant dislocation after THA. There was moderate heterogeneity across the studies (p = 0.18, I^2^ = 42%). No significant difference in the risk of implant dislocation was found between smokers and nonsmokers (summary RR = 1.27, 95% CI: 0.77–2.10) (Fig 4).

### All-cause revisions

**Fig 5:**
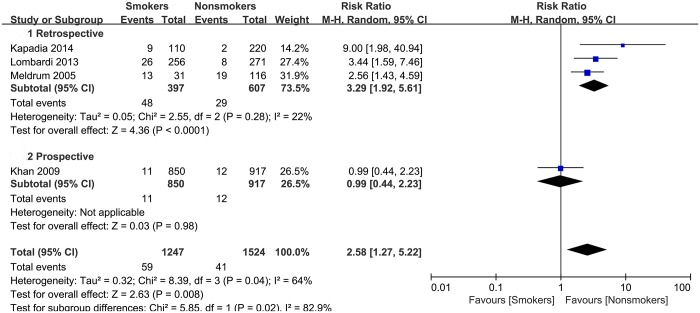
Forest plot of the association between smoking and the risk of all-cause revisions after total hip arthroplasty.

Revisions of the THA by exchange or removal of the components for any reason were documented in 4 studies with a total of 2771 patients. Because of the high heterogeneity among these studies (I^2^ = 64%), a random-effects model was employed. Compared with nonsmokers, smokers had a significantly increased risk of revisions after THA (summary RR = 2.58, 95% CI: 1.27–5.22). Subgroup analysis was performed for retrospective cohort studies. The heterogeneity was low across 3 retrospective studies (I^2^ = 22%), and smokers had a significantly higher risk than nonsmokers (summary RR = 3.29, 95% CI: 1.92–5.61) (Fig 5). Sensitivity analysis was also conducted, yielding similar results.

### Length of hospital stay

**Fig 6:**
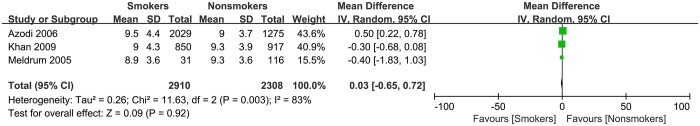
Forest plot of the association between smoking and the length of hospital stay after total hip arthroplasty.

Three studies, with a total of 5218 patients, were suitable for meta-analysis on the length of hospital stay after THA. A random-effects model was used due to the high heterogeneity across studies (I^2^ = 83%). The length of hospital stay did not differ significantly between smokers and nonsmokers following THA (WMD = 0.03, 95% CI: -0.65–0.72) (Fig 6). We excluded 1 study by Azodi et al. [13] that reported the median and quartiles, and the heterogeneity was eliminated (I^2^ = 0). However, no significant difference was observed (WMD = -0.31, 95% CI: -0.68–0.06).

## Discussion

In our meta-analysis of 6 cohort studies, smokers had a significantly higher risk of aseptic loosening of prosthesis, deep infection and all-cause revisions after THA than the patients who never smoked. However, no association was observed between smoking and the risk of implant dislocation or length of hospital stay after surgery.

Aseptic loosening of prosthesis is one of the most common complications, which can result in THA failure. In this meta-analysis, 3 studies were used to evaluate the risk of aseptic loosening of prosthesis after THA in the smokers. We excluded 1 study that lacked raw data, which demonstrated that agriculture work rather than smoking or other lifestyle features had a significant relationship with prosthetic loosening based on adjusted RR [20]. In this study, there were only 18 patients in the smoking group, whereas the nonsmoking group had 133 patients. The sample size of the smoking group was too small, which may greatly reduce the confidence in the results. Moreover, after we incorporated the adjusted RR in the meta-analysis, there was high heterogeneity (I^2^ = 66%). Therefore, this study should not be used. According to the outcomes of the present meta-analysis, we found that smoking was associated with a 2.05-fold increased risk of aseptic loosening of prosthesis after THA. It has been proposed that a negative balance between the rate of bone growth and bone resorption around the prosthesis results in the development of aseptic loosening [21]. Nicotine, the major effective ingredient of tobacco, can inhibit the secretion of tumor necrosis factor-α(TNF-α), which plays a pivotal role in the bone healing process as it promotes ossification by increasing chondrocyte apoptosis, osteoclast formation and matrix metalloproteinase expression as well as delays bone healing [22]. Moreover, many studies have demonstrated that smoking tobacco greatly impaired the osteointegration processes around the implanted biomaterials based on a coordinated cascade of complex events of cells and signal molecules [23]. However, until now, the exact mechanism of aseptic loosening of prosthesis is still inconclusive, and more investigations are required to explore it.

Deep infection after THA is a serious, even devastating, complication that requires surgical and long-term medical management [24,25]. Recently, a meta-analysis of 140 cohort studies, with a total of 479,150 participants, suggested a significantly higher risk of surgical site infection in smokers [26]. Therefore, it is reasonable to assume that smoking increases the risk of infection after THA. Indeed, our meta-analysis revealed a 2.71-fold increased risk of deep infection in the smokers undergoing THA. Even in the patients who had quit smoking, there was still a 1.97-fold increase in the RR of deep infection. Fender et al. [27] reported a rate of deep infection of 1.4% in a cohort of 1080 patients undergoing primary THA, which is similar to the overall rate (1.5%) in our meta-analysis. However, the overall rate in the cohort of smokers was 2.4%, which is much higher than the average rate. Various mechanisms have been proposed to elucidate the association between smoking and infection risk. Several investigations have demonstrated that a reduction of the tissue glucose and relative acidosis, attributed to the decreased tissue blood flow and oxygen in the smokers, resulted in an increased incidence of infection [28–30]. In addition, the adverse effect of cigarette smoke on the immune system contributes to the increased risk of infection [31].

In addition to aseptic loosening of prosthesis and deep infection, a variety of other complications, such as implant dislocation, prosthesis instability, liner breakage and periacetabular fracture, lead to the failures of THA. In the present meta-analysis, no association was found between smoking and the risk of implant dislocation, which is correlated with the BMI [12] and alcohol consumption [32]. However, the risk of revision for any reason increased by 1.58-fold in the smokers. Subgroup analysis of the retrospective cohort studies yielded similar results, and sensitivity analysis confirmed the stability of the positive association between smoking and the risk of all-cause revisions.

Although a higher risk of postoperative complications was reported in the smokers [13], there was no significant difference in the length of hospital stay between smokers and nonsmokers. Moller et al. [3] demonstrated that the mean length of hospital stay was similar between smokers and nonsmokers, and it was only prolonged in the smokers with postoperative complications. To the best of our knowledge, the number of patients with complications is small, and their length of hospital stay may not significantly alter the mean length of hospital stay of the entire cohort. Moreover, other confounding factors, such as the BMI and comorbidities of the patients, also greatly affect the length of hospital stay [13]. Therefore, it is not surprising that smokers and nonsmokers have a similar length of hospital stay. Furthermore, to eliminate high heterogeneity, we excluded a study that did not report the mean and standard deviation, and instead reported the median and quartiles, which may contribute to the high heterogeneity. However, the meta-analysis of 2 studies lacked a significant difference.

According to the study by Moller et al. [7], smoking cessation intervention significantly reduced wound-related complications, cardiovascular complications and the length of hospital stay in the patients undergoing total hip and knee replacement. Most recently, a meta-analysis by Thomsen et al. [33], based on 13 randomized control studies, also revealed the effectiveness of preoperative smoking cessation interventions on controlling postoperative complications. However, all of these investigations were based on short-term smoking interventions. As some prosthesis-related complications occur several years after THA, it is essential to completely quit smoking before and after surgery to avoid or postpone the occurrence of complications that cause THA failures. However, in spite this, former smokers still had a higher risk of prosthesis-related complications than nonsmokers in our meta-analysis. Hence, we should help more young people realize the harm of smoking and prevent them from becoming smokers.

There are some potential limitations in this investigation. First, the studies involved in the meta-analysis were all cohort studies, and observational studies can only provide information about correlations; but they do not reveal causality. Second, the definitions of smoking status are diverse and ambiguous. For example, in the study by Meldrum et al. [14], the authors did not provide a definition of smokers, and 5 smokers who had given up smoking after THA were also included in the study. Moreover, Khan et al. [11] defined former smokers as the patients who quit smoking over 30 days before admission to hospital, whereas former smokers in the study by Kapadia et al. [9] were labeled as the patients who quit smoking over 30 days before the date of the operation. Lombardi et al. [10] did not provide a definition for former smokers in their study. These characteristics may result in an under- or over-estimation of the risk. Third, the duration of follow-up varied, ranging from 2 months to 13 years. Short-term follow-up may underrate the risk of some complications, which may take place a few years after THA. Fourth, because most studies only performed univariate analyses, residual confounding is a limitation of these studies. However, the authors attempted to minimize confounding using matching or restriction methods at the study design stage. Most studies lacked a significant difference in the baseline characteristics of the study cohort. Although multivariate analysis was performed in some studies, the authors did not report intact adjusted values for the meta-analysis. Therefore, they cannot be incorporated in the present meta-analysis. However, the outcomes of the multivariate analysis reported in the individual studies were consistent with those of the present meta-analysis based on the studies that conducted univariate analyses. Fifth, there was substantial heterogeneity among studies. However, sensitivity analysis identified the source of heterogeneity and demonstrated the stability of our findings. Finally, the small number of available studies is a potential limitation. Our findings may be modified when more studies are performed.

## Conclusions

The present meta-analysis suggests that smoking is associated with significantly increased risks of aseptic loosening of prosthesis, deep infections and all-cause revisions after THA, but smoking does not increase the risk of implant dislocation or length of hospital stay. More well-designed investigations, both epidemiological and mechanistic, are required to validate our findings.

## Supporting Information

S1 PRISMA Checklist (DOC)

S1 Table Methodological quality of the studies included in the meta-analysis. (DOC)
