# Supplementary material for: Smoking and Risk of Prosthesis-Related Complications after Total Hip Arthroplasty: A Meta-Analysis of Cohort Studies
Source: PLoS One. 2015 Apr 24;10(4):e0125294. doi: 10.1371/journal.pone.0125294 (PMC4409354; doi:10.1371/journal.pone.0125294)
Supplement: S1 Table — (DOC) [file pone.0125294.s002.doc]

**Table S1.** Methodological quality of the studies included in the meta-analysis

| **Study** | **Selection** |  |  |  | **Comparability** | **Outcome** |  |  | **Total scores** |
| --- | --- | --- | --- | --- | --- | --- | --- | --- | --- |
|  | **Representativeness of the exposed cohort** | **Selection of the non-exposed cohort** | **Ascertainment of exposure** | **Outcome of interest not present at start of study** | **Control for important factors or additional factors** | **Outcome assessment** | **Follow-up long enough for outcomes to occur** | **Adequacy of follow-up of cohorts** |  |
| Kapadia et al. [9], 2014 | ☆ | ☆ | ☆ | - | ☆ | ☆ | ☆ | ☆ | 7 |
| Lombardi et al. [10], 2013 | ☆ | ☆ | ☆ | - | ☆ | ☆ | ☆ | ☆ | 7 |
| Khan et al. [11], 2009 | ☆ | ☆ | ☆ | ☆ | ☆ | ☆ | ☆ | ☆ | 8 |
| Azodi et al. [12], 2008 | ☆ | ☆ | ☆ | ☆ | ☆☆ | ☆ | ☆ | ☆ | 9 |
| Azodi et al. [13], 2006 | ☆ | ☆ | ☆ | - | ☆☆ | ☆ | - | ☆ | 7 |
| Meldrum et al. [14], 2005 | ☆ | ☆ | - | - | ☆ | ☆ | ☆ | ☆ | 6 |
